# The “*Taygetis ypthima* species group” (Lepidoptera, Nymphalidae, Satyrinae): taxonomy, variation and description of a new species

**DOI:** 10.3897/zookeys.356.6481

**Published:** 2013-11-27

**Authors:** Ricardo Russo Siewert, Thamara Zacca, Fernando Maia Silva Dias, André Victor Lucci Freitas, Olaf Hermann Hendrik Mielke, Mirna Martins Casagrande

**Affiliations:** 1 Laboratório de Estudos de Lepidoptera Neotropical, Departamento de Zoologia, Universidade Federal do Paraná, P.O. Box 19.020, ZIP Code 81.531-980, Curitiba, Paraná, Brazil; 2 Departamento de Biologia Animal and Museu de Zoologia, Instituto de Biologia, Universidade Estadual de Campinas, P.O. Box 6.109, ZIP Code 13.083-970, Campinas, São Paulo, Brazil

**Keywords:** Atlantic Forest, Euptychiina, Neotropical, *Pseudodebis*, *Taygetis rectifascia*

## Abstract

A new species of *Taygetis* Hübner, [1819] (Lepidoptera, Nymphalidae, Satyrinae) from southeastern Brazil is described: *Taygetis drogoni* **sp. n.** In addition, *T. servius* Weymer, 1910 and *T. fulginia* d’Almeida, 1922 are resurrected from synonymy and a taxonomic discussion on the species *T. ypthima* Hübner, [1821] and *T. rectifascia* Weymer, 1907 is provided. A dichotomous key for the species is also provided.

## Introduction

The butterfly subtribe Euptychiina includes over 400 described species in 45 genera, being one of the most diverse groups in the subfamily Satyrinae (Lepidoptera, Nymphalidae) (Lamas 2004, Peña et al. 2010, Zacca et al. 2013). This group is predominantly Neotropical, but some species also occur in the Neartic region and in Southeast Asia (Peña et al. 2010, Matos-Maraví et al. 2013). The taxonomy of the group is among the most poorly known of all Neotropical butterflies, and the relationships within the subtribe are still in debate (Marín et al. 2011). Several genera are polyphyletic, new species and genera are regularly described, and the delimitation of the recognized genera and species needs much effort before a better understanding on the systematics of the group emerges (e.g. Freitas and Peña 2006, Freitas et al. 2011, in press, Zacca et al. 2013).

In a recent study based on molecular data, Peña et al. (2010) proposed a phylogenetic hypothesis of Euptychiina, and defined five major clades within this group. The “*Taygetis* clade” is one of those five major groupings and it includes 10 valid genera. A preliminary molecular phylogeny of the “*Taygetis* clade” (Matos-Maraví et al. 2013) gave insights on the non-monophyletic nature of most genera, and also revealed several new cryptic species waiting to be described. *Taygetis* appeared as polyphyletic, with *Taygetis kerea* Butler, 1869 and *Taygetis weymeri* Draudt, 1912 as part of the “*Taygetina* subclade”, and with the clade *Taygetis ypthima*+*Taygetis rectifascia* (hereafter “*Taygetis ypthima* species group”), as part of the “*Pseudodebis* subclade” (Matos-Maraví et al. 2013).

The genus *Taygetis* comprises 27 described species and several undescribed species (Lamas 2004), which are widely distributed throughout the neotropics, from Mexico to Uruguay (Lamas 2004, Marín et al. 2011). Adult *Taygetis* are mid-sized to large butterflies, with brown dorsal wings and with the ventral surface resembling dried leaves (D’Abrera 1988). Some species are crepuscular and are easily captured using rotting fruit in bait traits (Murray 2001a, b). A number of species of *Taygetis* show high apparent intraspecific phenotypic variation, and some species have been described several times. For example, *Taygetis virgilia* (Cramer, 1776) has five synonymized names (Lamas 2004).

Although intraspecific phenotypic variation appears to be common in several Euptychiina, in some cases hidden taxonomic diversity might be underestimated. Similar to *Taygetis virgilia* (see above), *Taygetis ypthima* is a highly variable species that has five synonymized names (Lamas 2004).

The present paper studied in detail the morphology of male and female genitalia and wing pattern variation of *Taygetis ypthima*, and related species, such as its sister species *Taygetis rectifascia* Weymer, 1907 (Matos-Maraví et al. 2013). As a result, a new species of *Taygetis* from Brazil is described, and *Taygetis servius* Weymer, 1910 and *Taygetis fulginia* d’Almeida, 1922, synonyms of *Taygetis rectifascia* and *Taygetis ypthima* respectively (see Lamas 2004), were revalidated.

## Methods

Dissections of the genitalia were made following standard techniques. The abdomen was removed, soaked in a heated 10% KOH solution for 5 minutes before dissection of the genitalia to analyze its structures. Illustrations were prepared with the aid of a camera lucida attached to a stereoscopic microscope. Genitalia terminology follows Oiticica-Filho (1946) and Niculescu (1972-1983). Distributional data were obtained from seven entomological institutions (see below) and, when possible, from the literature (Biezanko 1960, Ebert 1969, Kochalka et al. 1996, Krüger and Silva 2003, Iserhard and Romanowski 2004, Quadros et al. 2004, Uehara-Prado et al. 2004, Giovenardi et al. 2008, Paz et al. 2008, Bonfantti et al. 2009, Iserhard et al. 2010, Peña et al. 2010, Dolibaina et al. 2011, Pedrotti et al. 2011, Santos et al. 2011, Soares et al. 2011, Bellaver et al. 2012, Silva et al. 2012). Ventral and dorsal wings surfaces were photographed and their patterns compared to original descriptions. All previously described taxa were studied in detail, including photographs of type specimens and original descriptions. Dissections were made for individuals corresponding to all observed variation, including phenotypes corresponding to all available names for the species in this group.

All examined material belongs to the following institutions:

DZUP Coleção Entomológica Padre Jesus Santiago Moure, Curitiba, Paraná, Brazil

UFMG Universidade Federal de Minas Gerais taxonomic collection, Belo Horizonte, Minas Gerais, Brazil

ZUEC Museu de Zoologia Adão José Cardoso, Universidade Estadual de Campinas, Campinas, São Paulo, Brazil

ZUEC-AVLF André VL Freitas Collection, Universidade Estadual de Campinas, Campinas, São Paulo, Brazil

SMFL
Lepidoptera collection, Senckenberg-Museum, Frankfurt am Main, Germany

SMT Staatliches Museum für Tierkunde, Dresden, Germany

ZSM Zoologische Staatssammlung München, Germany

## Results

### Taygetis drogoni

Siewert, Zacca, Dias & Freitas sp. n.

http://zoobank.org/CA6CEBB1-A525-44EE-A265-CE467707BFB5

http://species-id.net/wiki/Taygetis_drogoni

**Figure 1.**
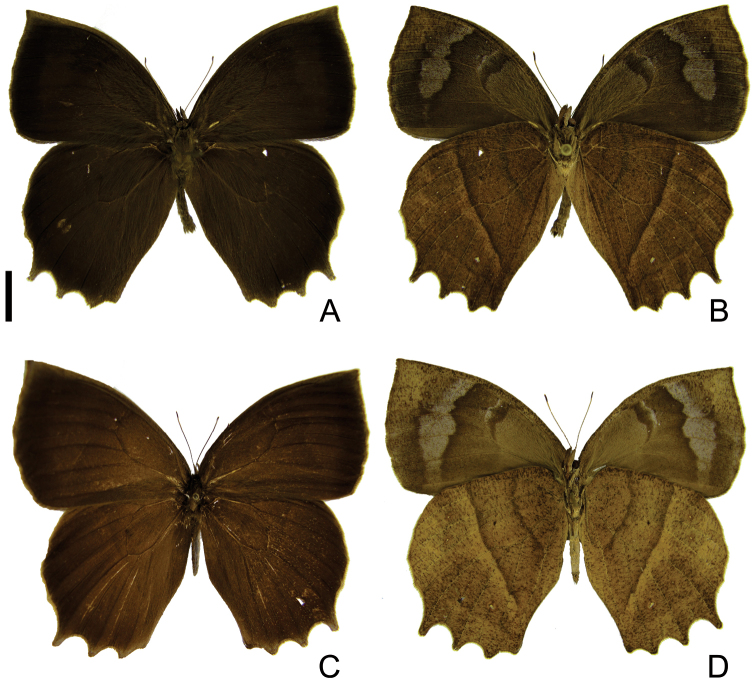
Adults of *Taygetis drogoni* sp. n. **A–B** male **A** dorsal view **B** ventral view **C–D** female **C** dorsal view **D** ventral view. Scale bar = 1 cm.

Figs 1A–D ; 6A–E ; 7A, B

#### Type material.

Holotype male with the following labels (separated by transverse bars): /HOLOTYPUS/ *Taygetis drogoni* Siewert, Zacca, Dias & Freitas det. 2013/ M(#)/ 07-II-1985 Cambuquira, M[inas] G[erais] [21°51'30"S, 45°17'28"W]. Mielke & Casagrande leg./ DZ 27.604/ (DZUP). Allotype female with the following labels (separated by transverse bars): /ALLOTYPUS/ *Taygetis drogoni* Siewert, Zacca, Dias & Freitas det. 2013/ F(#)/ 10-XII-1968 Camb[uquira], [Minas Gerais] [21°51'30"S, 45°17'28"W]./ Coleção H. Ebert/ DZ 27.607/ (DZUP).

#### Paratypes.

BRAZIL – *Minas Gerais*: **Alfenas** – 14-XII-2011, 1 female, Brito leg., JCI2.1-130 (ZUEC); 08-II-2012, 1 male, Brito leg., JCI3.2-225 (ZUEC). **Cambuquira** – 6-X-1968, 1 female, Ebert leg., ex-coll. Ebert, DZ 5.501 (DZUP); 10-XII-1968, 6 males, Ebert leg., ex-coll. Ebert, DZ 27.606, DZ 27.607, DZ 27.614, DZ 27.619, DZ 27.623, DZ 27.624 (DZUP); 900 m, 15-IV-1969, 3 males, Ebert leg., ex-coll. Ebert, DZ 27.605, DZ 26.419, DZ 27.620 (DZUP); 2-7-XI-1969, 1 female, Ebert leg. (SMFL); 15-V-1981, 1 male, Ebert leg., ex-coll. Ebert, DZ 27.618 (DZUP); 7-II-1985, 5 males, Mielke & Casagrande leg. DZ 27.621, DZ 27.431, DZ 27.616, DZ 27.626, DZ 27.627 (DZUP). **Caraça** - Santa Barbara, 1500 m, 1-5-II-1985, 1 male and 1 female, Mielke & Casagrande leg. DZ 27.625, DZ 27.629 (DZUP). **Carmo do Rio Claro** – 1-VIII-1948, 1 male, Carvalho & Alceu leg. DZ 27.617 (DZUP). **Nova Lima** – APE Manancial Mutuca, Parque Estadual da Serra do Rola Moça, 1-V-2009, 1 male, Silva leg., DNA voucher PM 10-02 (ZUEC-AVLF). **São Roque de Minas** – Parque Nacional Serra da Canastra, 9-IV-1999, 1 male, without collector, UFMG ILE 1300506 (UFMG); 19-21-IV-1999, 2 males, without collector, UFMG ILE 1300504, 1300507 (UFMG). *São Paulo*: **São Luiz do Paraitinga** – 800 m, 22-IV-2004, 1 male, Ribeiro leg., ZUEC LEP 6.548 (ZUEC); 28-IX-2004, 1 male, Ribeiro leg., ZUEC LEP 6.548 (ZUEC); 29-IX-2004, 1 male, Ribeiro leg., ZUEC LEP 7.003 (ZUEC); 12-I-2005, 1 female, Ribeiro leg., ZUEC LEP 6.724 (ZUEC); 18-II-2005, 2 males, Ribeiro leg., ZUEC LEP 6.666, ZUEC LEP 6.691 (ZUEC).

#### Diagnosis.

*Taygetis drogoni* sp. n. is very similar to *Taygetis ypthima*, differing from the latter by the following characters: forewing with pale brown dorsal post discal band less contrasting than in *Taygetis ypthima*; underside pale post discal band slightly constricted at M_3_, tapering abruptly in CuA_1_-CuA_2_ and becoming conspicuously thinner or even absent from CuA_2_ to the inner margin; hind wing underside with the discal line evenly curved and regular, extending from the costal margin to 1A; and dark post discal line straight and only slightly irregular. Tegumen larger and protruding; valva stouter and shorter, with a larger dorsal rough area. Signa dorsal; laterally, sternum VIII not fused with tergum VIII; lamella antevaginalis without process.

#### Description.

*Head*. Brown. Post-genal area light brown. Eye glabrous, brown. Antennae without scales at apical third, mostly light brown; club dark brown with last flagellomere light brown. Labial palpus mixed with brown and light brown, with elongated scales at first and second segment; about 1.5 times total length of eye; third segment thin, same size as first. *Thorax*. Uniformly brown. Legs brown; meso- and metathoracic femurs light brown on inside. *Forewing, size and shape*: length: 34.5–37.0 mm (n = 23). Triangular, costal margin convex, apex pointed, outer margin convex, tornus rounded, inner margin straight. *Forewing upper side* (Fig. 1A, C). Mostly brown, darker along outer margin. *Forewing under side* (Figs 1B, D). Background brown, lighter at wing base. Dark brown scales at end of discal cell and whitish on transverse veins. Dark spot at base of M_2_. Costal margin to external margin with reticulated markings. Apex rufous brown. Submarginal band whitish, from costal to inner margin, with reduced creamy ocelli in spaces R_5_-M_1_, M_1_-M_2_, M_2_-M_3_ and M_3_-CuA_1_; proximal border of submarginal band irregular with dark brown scaling, distal border of marginal band inconspicuous. Marginal line brown. Fringe light brown. *Hind wing shape*: Costal margin convex, apex rounded, external margin convex in M_1_-M_2_, projections at CuA_1_, CuA_2_ and 2A, with a developed one at M_3_. Inner margin curved at base, slightly straight towards tornus. *Hind wing upper side* (Fig. 1A, C). Mostly brown, darker along outer margin. *Hind wing under side* (Fig. 1B, D). Background rufous brown, discal line dark brown and irregular. Dark brown spot at base of M_2_. Submarginal band with reduced creamy ocelli, in spaces Rs-M_1_, M_1_-M_2_, M_2_-M_3_ and M_3_-CuA_1_, ocellus on CuA_1_-CuA_2_ developed. Post discal line straight and dark brown. Proximal border of submarginal band along post discal line forming a 2 mm wide reddish fascia. Distal border of marginal band inconspicuous. Marginal line brown, with distal area reddish. Fringe light brown. *Abdomen*. Dorsally brown, ventrally light brown.

**Male genitalia** (Fig. 6A–E). Tegumen dorsally convex, subtriangular in lateral view, ventral projection wide; appendix angular reduced. Uncus straight, down curved at apex and dorsally keeled. Gnathos larger than uncus; straight and projected dorsally, without a ventral projection at base. Anterior projection of saccus cylindrical, length equal to tegumen. Valva subrectangular, with dorsal projections at apical third; costa developed and subtriangular; ventrally covered by setae. Aedeagus straight, thin and larger than valva; opening of aedeagus almost the total length of posterior portion.

**Female genitalia** (Fig. 7A, B). Tergum VIII squared. Papillae anales with setae at distal portion, 2/3 higher than longer, sclerotized at basal half. Sterigma sclerotized, formed by a round lamella antevaginalis and a membranous pocket between ostium bursae and papilla anales. Bursa copulatrix totally membranous, with a pair of signa dorsally; ductus bursae thinned, with apical third sclerotized, about three times length of bursa copulatrix.

#### Etymology.

The specific epithet refers to Drogon, one of the three dragons of Daenerys Targaryen, a fictional character from the George R. R. Martin’s novel “A Song of Ice and Fire”.

#### Distribution

(Fig. 8). This species occurs in southeastern Brazil (Minas Gerais and São Paulo) at elevations from 800 to 1,500 m a.s.l.

#### Taxonomic comments.

This species has presumably not been recognized in the past because of the intrinsic phenotypic variation within *Taygetis* and, in particular, within *Taygetis ypthima*, the most similar and probably closest species to *Taygetis drogoni* sp. n. The species appears cited as *Taygetis ypthima* in Ribeiro et al. (2012). In Matos-Maraví et al. (2013), *Taygetis ypthima* PM10-02 is in fact *Taygetis drogoni* (one of the paratypes, see above). A real *Taygetis ypthima* (*Taygetis ypthima* NW 149-8) is also included in that study, and it appears as sister to *Taygetis drogoni* in the phylogeny presented in that paper. The genetic distances between these two specimens in the available COI and nuclear genes provide further support for the description of *Taygetis drogoni* sp. n.

### Taygetis ypthima

Hübner, [1821]

http://species-id.net/wiki/Taygetis_ypthima

**Figure 2.**
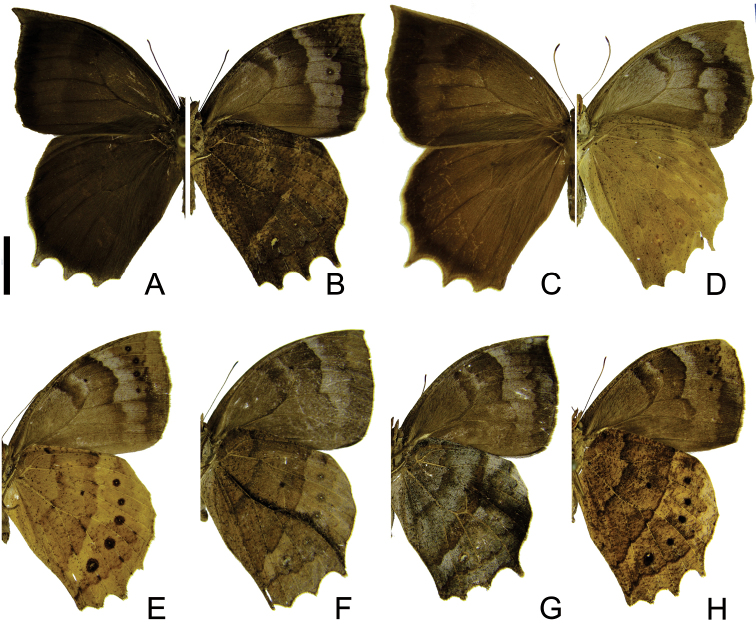
Adults of *Taygetis ypthima*. **A–B** male **A** dorsal view **B** ventral view **C–D** female **C** dorsal view **D** ventral view **E–H** variations in ventral view. Scale bar = 1 cm.

Figs 2A–H ; 6F–J ; 7C, D

#### Examined material.

BRASIL – *Bahia*: **Jitaúna** – 25-III-1969, 1 female, Ebert leg., ex-coll. Ebert, DZ 27. 720 (DZUP). Rio de Contas, 150 m, 4-III-1969, 1 female, H. Ebert leg., ex-coll. Ebert, DZ 27.721 (DZUP). *Minas Gerais*: **Alfenas** – 27-I-2012, 1 male, Brito leg., VPI2.2-214 (ZUEC). **Camanducaia** – Monteverde, 1650m, 8-II-1979, Ebert leg., ex-coll. Ebert, DZ 27.646 (DZUP); **Cambuquira** – 10-XII-1968, 1 male and 1 female, Ebert leg., ex-coll. Ebert, DZ 27.432, DZ 26.433 (DZUP); 12-X-1968, 1 female, Ebert leg., ex-coll. Ebert, DZ 26.434 (DZUP); 15-IX-1969, 1 female, Ebert leg., ex-coll. Ebert, DZ 27.647 (DZUP); **Conceição dos Ouros** – Rio Sapucaí, 24-II-1968, 1 female, Ebert leg., ex-coll. Ebert, DZ 27.648 (DZUP); **Marliéria** – Parque Estadual do Rio Doce, 200 m, 08-IX-1972, 1 female, Ebert leg., ex-coll. Ebert, DZ 27.718 (DZUP); 16-III-1972, 1 male, H. & H. D. Ebert leg., ex-coll. Ebert, DZ 27.719 (DZUP). **Itamonte** – Vargem Grande, 1600m, 17-II-2010, 1 male, Mielke & Casagrande leg., DZ 27.554 (DZUP); NE side of Itatiaia, 1300m, II-1959, 1 male, Ebert *leg*., ex-coll. Ebert, DZ 27.671 (DZUP); **Passa Quatro** – Paiolinho, Fazenda Serra Fina, 1600m, 16-II-2010, 1 male, Mielke & Casagrande leg., DZ 27.645 (DZUP); **Virgínia** – Fazenda dos Campos, 1500m, 13-15-II-2010, Mielke & Casagrande leg., DZ 27.540 (DZUP). *Rio de Janeiro*: **Nova Friburgo** – 1000m, 23-I-1983, 1 female, O.-C. Mielke leg., DZ 27.644 (DZUP); **Itatiaia** – 900m, 23-I-1936, 1 male, Gagarin leg., ex-coll. Gargarin, DZ 27.472; II-1958, 2 male, Ebert leg., ex-coll. Ebert, DZ 27.634, DZ 27.635 (DZUP); 1600m, 14-II-1956, 1 male and 2 females, Ebert leg., ex-coll. Ebert, DZ 27.637, DZ 27.638, DZ 27.639 (DZUP); Parque Nacional do Itatiaia, Maromba, 1100m, 06-09-II-2011, 1 female, Freitas leg., ZUEC LEP 5.372 (ZUEC); **Petrópolis** – Independência, 900m, 13-III-1933, 1 male, Gargarin leg., ex-coll. Gagarin, DZ 27.655 (DZUP); **Rio de Janeiro** – 14-XI-1920, 1 male, D’Almeida leg., ex-coll. D’Almeida, DZ 27.654 (DZUP); **Teresópolis** – 1600m, 20-II-1967, 1 female, Ebert *leg*., ex-coll. Ebert, DZ 27.637 (DZUP). *São Paulo*: **Apiaí** – IV-1972, 1 male, Ebert leg., ex-coll. Ebert, DZ 27.636 (DZUP); **Campos do Jordão** – I.1966, 5 males and 3 females, without collector, DZ 27.369, DZ 27.579, DZ 27.588, DZ 27.589, DZ 27.591, DZ 27.597, DZ 27.602, DZ 27.603 (DZUP); 1600-2000m, 8-12-II-1982, 2 males, Mielke & Casagrande, DZ 27.587, DZ 27.595 (DZUP); Parque Estadual Campos do Jordão, 1950m, 10-II-1968, 2 males, Mielke, Brown & Laroca leg., DZ 27.593, DZ 27.600 (DZUP); 1800m, 11-12-I-2001, 1 female, Brown & Freitas leg. (ZUEC-AVLF); **Capão Bonito** – Fazenda Intervales, Sede, 950-1100m, 30-XII-1989, 1 female, Freitas leg. (ZUEC-AVLF); 15-II-2000, 2 females, Brown, Freitas, Francini & Uehara-Prado leg., ZUEC LEP 1.776 (ZUEC); 13-XII-2000, 1 male and 1 female, Brown, Freitas, Francini & Uehara-Prado leg., ZUEC LEP 4.731, ZUEC LEP 4.732 (ZUEC); 5-6-XII-2001, 1 male and 1 female, Brown & Freitas leg. (ZUEC-AVLF); 15-I-2002, 1 female, Brown, Freitas, Francini & Uehara-Prado leg., ZUEC LEP 802 (ZUEC); 17-I-2003, 2 males and 2 females, Brown, Freitas, Francini & Uehara-Prado leg., ZUEC LEP 1.028, ZUEC LEP 1.547, ZUEC LEP 1.548, ZUEC LEP 1.549 (ZUEC); 19-I-2003, 3 males, Brown, Freitas & Uehara-Prado leg., ZUEC LEP 1.180, ZUEC LEP 1.286, ZUEC LEP 1.305 (ZUEC); **Cotia** – Morro Grande, 900-1100m, 15-III-2000, 1 female, Uehara-Prado & Freitas leg. (ZUEC-AVLF); 22-XII-2000, 1 female, Brown & Uehara-Prado leg. ZUEC LEP 1.781 (ZUEC); **Imbariê** – 7-I-1956, 1 female, Ebert leg., ex-coll. Ebert, DZ 27.633 (DZUP); **Jundiaí** – Serra do Japi, 11-V-2012, 1 female, Santos leg., BLU 246 (ZUEC); **Piquete** – Barreira de Piquete, 1400-1600m, 15-II-1984, 2 males, Mielke & Casagrande *leg*., DZ 27.585, DZ 27.596 (DZUP); **Presidente Venceslau** – without date, 2 males and 4 females, D’Almeida leg., DZ 27.656, DZ 27.657, DZ 27.658, DZ 27.659, DZ 27.660, DZ 27.661 (DZUP); **Rio Claro** – 60m, 6-I-1964, 5 males and 1 female, Ebert leg., ex-coll. Ebert, DZ 27.392, DZ 5.500, DZ 27.663, DZ 27.630, DZ 27.631, DZ 27.632 (DZUP); 16-V-1965, 1 male, Ebert leg., ex-coll. Ebert, DZ 27.662 (DZUP); **Salesópolis** – Estação Biológica da Boraceia, 900m, 21-II-2006, 1 male, Uehara-Prado & Freitas leg. (ZUEC-AVLF); 27-III-2006, 1 male, Uehara-Prado & Freitas leg. (ZUEC-AVLF); **Umuarama** – 1800m, 8-15-III-1937, 8 males and 2 females, Gargarin leg., ex-coll. Gagarin, DZ 27.584, DZ 27.586, DZ 27.590, DZ 27.592, DZ 27.593, DZ 27.594, DZ 27.598, DZ 27.599, DZ 27.601, DZ 27.632 (DZUP). *Paraná*: **Foz do Iguaçu** – 250m, 17-II-1969, 3 males, Moure & Mielke leg., DZ 26.741, DZ 1.626, DZ 27.368 (DZUP); 10-X-1969, 1 male, Krause leg., DZ 26.740 (DZUP); Parque Nacional do Iguaçu, 21-24-IV-1996, 2 females, Mielke & Casagrande leg., DZ 26.743, DZ 26.769 (DZUP); **Curitiba** – 900m, 20-III-1980, 1 male, O. Mielke leg., DZ 27.405 (DZUP); Uberaba, Tirol das Torres, 900m, 5-II-2010, 1 female, O. Mielke leg., DZ 26.742 (DZUP); **Rolândia** – Rio Tibagi, 750m, XII-1941, 1 male, Waltz leg., DZ 27.397 (DZUP). *Santa Catarina*: **Canoinhas** – I, 1 male, Pohl *leg*., DZ 27.667 (DZUP); 16-IX-1941, 1 male, Schimith *leg*., DZ 27.668 (DZUP); **Ibirama** – I, 1 male, Pohl leg., DZ 27.666 (DZUP); VIII, 1 female, Pohl *leg*., DZ 27.670 (DZUP); XII, Pohl *leg*., DZ 27.669 (DZUP); **Itaiópolis** – 26-III-1937, 1 male, D’Almeida leg., ex-coll. D’Almeida, DZ 27.549 (DZUP); **Itajaí**– Agrolândia, 400m, II-1973, 1 female, Wulff leg., DZ 27.665 (DZUP); **Joinville** – 5-III-1974, 1 male, O. Mielke leg., DZ 26.778 (DZUP); **São Bento do Sul** – Rio Vermelho, 850m, 10-IV-1980, 1 female, Rank leg., DZ 27.640 (DZUP); 950m, 23-I-1982, 1 male, Rank leg., DZ 26.815 (DZUP); 650m, 30-I-1982, 1 male, Rank leg., DZ 26.816 (DZUP); 850m, 7-XII-1969, 1 male, Ebert leg., ex-coll. Ebert, DZ 27.649 (DZUP); 8-I-1971, Ebert leg., ex-coll. Ebert, DZ 27.643 (DZUP); 10-I-1971, 1 female, Ebert leg., ex-coll. Ebert, DZ 27.641 (DZUP); 7-VIII-1971, Ebert leg., ex-coll. Ebert, DZ 27.650 (DZUP); 3-X-1971, 1 female, Ebert leg., ex-coll. Ebert, DZ 5.499 (DZUP); 5-XII-1969, 1 male and 1 female, Ebert leg., ex-coll. Ebert, DZ 27.642, DZ 27.651 (DZUP); 6-XII-1969, 1 male and 1 female, Ebert leg., ex-coll. Ebert, DZ 26.429, DZ 27.653 (DZUP); 4-III-1980, 1 male, Ebert leg., ex-coll. Ebert, DZ 27.652 (DZUP); 8-V-1980, 1 male, Rank *leg*., DZ 27.664 (DZUP); **Taió** – February, 1 male, Pohl *leg*., DZ 27.666 (DZUP). *Rio Grande do Sul*: **São José do Inhacorá** – Alto Uruguai, 2-V-1980, 1 male, Steiniger leg., DZ 26.770 (DZUP). PARAGUAY – *General Dias*: **Itaquiri** – 400m, 15-20-I-1980, 5 males and 5 females, O.-C. Mielke & Myers *leg*., DZ 27.708, DZ 27.709, DZ 27.710, DZ 27.711, DZ 27.712, DZ 27.713, DZ 27.714, DZ 27.715, DZ 27.716, DZ 27.717 (DZUP). ARGENTINA – *Corrientes*: **Santo Tomé** – I-1924, 1 male, D’Almeida leg., ex-coll. D’Almeida, DZ 27.522 (DZUP). *Misiones*: **General Manuel Belgrano** – Almirante Brown, Reserva Yacutinga, 2-5-III-2007, 1 male, Mielke & Casagrande leg., DZ 27.531 (DZUP). *Tucumán*: **Ibatim** – Pueblo Viejo, 850m, 25-I-1970, 1 female, O. Mielke leg., DZ 27.707 (DZUP).

#### Diagnosis.

*Taygetis ypthima* can be distinguished from *Taygetis drogoni* by the forewing underside submarginal band, not conspicuously constricted at M_3_; the proximal line is oblique in M_3_-CuA_1_ to the direction of the base of the wing, disjointed of the remainder of the line from CuA_1_ to the inner margin; submarginal band irregular, but about the same width from M_1_ to the inner margin, sometimes slightly wider at M_3_-CuA_1_; hind wing underside with the discal line curved and irregular, extending from the costal margin to the inner margin; and proximal line of the submarginal band distinctly curved and irregular. Tegumen smaller; valva thinner and longer, with a smaller dorsal rough area. Signa ventral; laterally, sternum VIII fused with tergum VIII; lamella antevaginalis with two lateral process.

#### Distribution

(Fig. 8). Occurs in northeastern, southeastern and southern Brazil, and also in Paraguay and Argentina, from sea level to 2000 m a.s.l. Based on label data, adults are present all year round.

#### Taxonomic comments.

This is the commonest and more widespread species of the group. The high intraspecific variation observed in *Taygetis yphtima* yield a number of descriptions of local forms or synonyms: *Taygetis xantippe* Butler, [1870], *Taygetis ophelia* Butler, 1870; *Taygetis ophelia* f. *semibrunnea* Weymer, 1910 and *Taygetis ypthima* [*sic*] ab. *lineata* Kivirikko, 1936, all synonyms of *Taygetis ypthima* (Lamas 2004; Warren et al. 2013). Based on collected specimens, different phenotypes associated with these taxonomic names frequently occur in a same locality. Furthermore, the genitalia of these specimens are exactly alike the genitalia of typical *Taygetis ypthima*. Nonetheless, *Taygetis fulginia* D’Almeida, 1922, until recently considered a synonym of *Taygetis ypthima* (Lamas *op. cit.*), is in fact a valid species, with clear differences on morphology of male and female genitalia and wing pattern (see below).

### Taygetis rectifascia

Weymer, 1907

http://species-id.net/wiki/Taygetis_rectifascia

**Figure 3.**
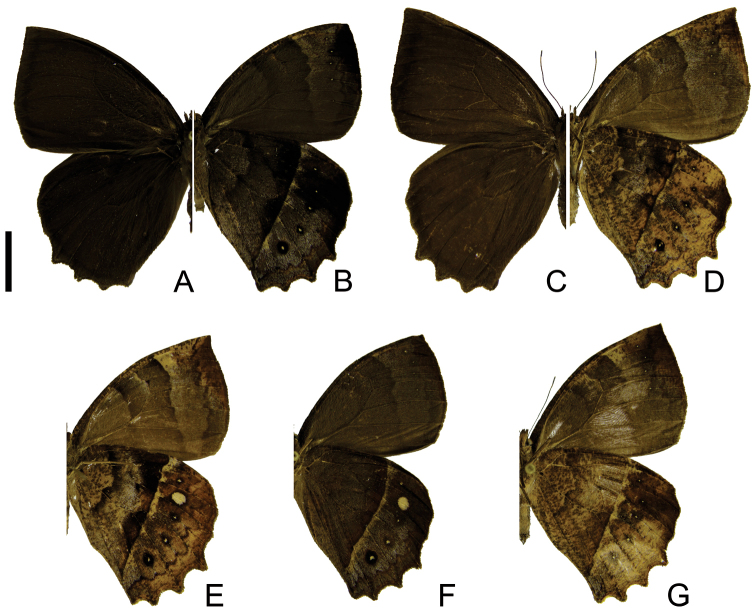
Adults of *Taygetis rectifascia*. **A–B** male **A** dorsal view **B** ventral view **C–D** female **C** dorsal view **D** ventral view **E–G** variations in ventral view. Scale bar = 1 cm.

Figs 3A–G ; 6K–O ; 7E, F

#### Examined material.

BRASIL – *Rio de Janeiro*: **Rio de Janeiro** – Mangaratiba - 12-VIII-1926, 1 female, D’Almeida leg., ex-coll. D’Almeida, DZ 26.426 (DZUP); **Itatiaia** - Parque Nacional do Itatiaia, 1200m, 25-II-1959, 1 male, Ebert leg., ex-coll. Ebert, DZ 27.490 (DZUP); 1000-1200m, 25-VII-1963, 1 male, Ebert leg., ex-coll. Ebert, DZ 26.417 (DZUP); 1100m, 4-II-1966, 1 male, Ebert leg., ex-coll. Ebert, DZ 27.469 (DZUP); 110m, 29-III-1967, Ebert leg., ex-coll. Ebert, DZ 5.502 (DZUP); 1000-1200m, 25-VII-1968, 1 male, Ebert leg., ex-coll. Ebert, DZ 27.346 (DZUP). *São Paulo*: **Capão Bonito** – Fazenda Intervales, Sede, 950m, 28-XII-1989, 1 male, Freitas leg. (ZUEC-AVLF); 30-XII-1989, 1 male, Freitas leg. (ZUEC-AVLF); 15-II-2000, 3 males and 3 females, Brown, Freitas, Francini & Uehara-Prado leg., ZUEC LEP 1.777, ZUEC LEP, 1.778, ZUEC LEP 1.779, ZUEC LEP 1.780, ZUEC LEP 1.782, ZUEC LEP 1.785 (ZUEC); 13-XII-2000, 3 males and 3 females, Brown, Freitas, Francini & Uehara-Prado leg., ZUEC LEP 4.730, ZUEC LEP 4.733,ZUEC LEP 4.734, ZUEC LEP 4.735, ZUEC LEP 4.736 (ZUEC); 5-6-XII-2001, 1 male and 4 females, Brown & Freitas leg. (ZUEC-AVLF); 17-20-I-2003, 3 males, Brown, Freitas & Uehara-Prado leg., ZUEC LEP 1.188, ZUEC LEP 1.189, ZUEC LEP 1.550 (ZUEC); **Salesópolis** – Estação Biológica da Boraceia, 900m, 28-IV-2006, 1 male, Uehara-Prado & Freitas leg. (ZUEC-AVLF); *Paraná*: **Campina Grande do Sul** – 13.III.1982, 2 males and 2 female, Mielke & Casagrande leg., DZ 26.430, DZ 27.519, DZ 27.532, DZ 27.534 (DZUP); *Santa Catarina*: **Taió** – I, 1 male, Pohl leg., DZ 27.382; **Presidente Getúlio** – Dalbérgia, 1 male, December, Pohl leg., DZ 26.425 (DZUP); **São Bento do Sul** – Rio Natal, IV-2012, 1 male, Rank leg., DZ 27.449 (DZUP).

#### Diagnosis.

*Taygetis rectifascia* can be distinguished from *Taygetis fulginia* and other species of the genus by the following combination of characters: forewing pointed at the apex; hind wing, with small projections at M_3_, CuA_1_ and CuA_2_; dorsal wings brown with thin suffused dark brown bands about 2 mm away from and along the outer margin; ventral hind wing with the proximal border of the submarginal band and post discal line straight and slightly irregular, sometimes forming a creamy white fascia of varying width. The base of the gnathos presents a ventral pointed projection, similar to *Taygetis fulginia* and *Taygetis servius* stat. n. The male genitalia differ from all other species discussed in the present paper by the shape of the valvar end, which is bifid and claw-shaped (presenting large intraspecific variation).

#### Distribution

(Fig. 8). Occurs in southeastern and southern Brazil (Rio de Janeiro, São Paulo, Paraná and Santa Catarina), at elevations from 300 to 1,200 m a.s.l. Based on label data, adults are present all year round.

#### Taxonomic comments.

Despite its superficial resemblance to *Taygetis fulginia*, this species presents a very distinctive genitalia. *Taygetis rectifascia* presents strong intraspecific variation in the wing pattern, which in the past has motivated the description of several aberrations and forms: *Taygetis rectifascia* ab. *stigma* Weymer, 1907; *Taygetis rectifascia* ab. *latifascia* Weymer, 1907 (all synonyms of *Taygetis rectifascia*) (Warren et al. 2013). *Taygetis epithyma* Forster, 1964 is a *nomem nudum*. The genitalia of these different phenotypes are alike the genitalia of typical *Taygetis rectifascia*.

### Taygetis servius

Weymer, 1910 stat. n.

**Figure 4.**
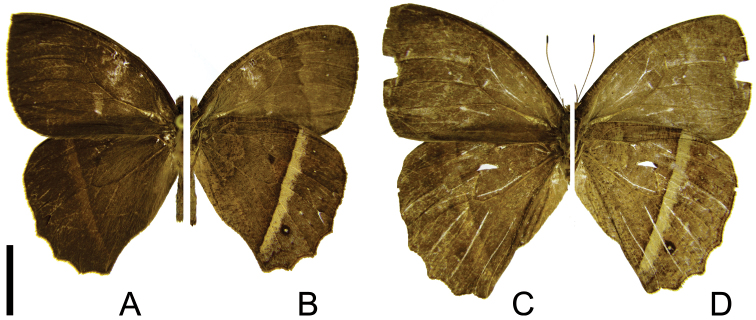
Adults of *Taygetis servius* stat. n. **A–B** male **A** dorsal view **B** ventral view **C–D** female **C** dorsal view **D** ventral view. Scale bar = 1 cm.

Figs 4A–D , 6P-T ; 7G–H

#### Examined material.

BRAZIL – *Bahia*: **Jitaúna** – 26-III-1969, 2 males and 2 females, Ebert leg., ex-coll. Ebert, DZ 26.424, DZ 27.440, DZ 27.396, DZ 27. 386 (DZUP). *Espírito Santo*: **Baixo Guandú** – 10-IV-1970, 1 male, Elias leg., DZ 26.806 (DZUP).

#### Diagnosis.

*Taygetis servius* stat. n. can be distinguished from *Taygetis fulginia* stat. r. and *Taygetis rectifascia* by the following characters: forewing rounded at the apex; dorsal wings light brown without any suffused dark brown bands along the outer margin; ventral hind wing with the proximal border of the submarginal band and post discal line straight and regular, forming a 2 mm wide creamy white fascia. The base of the gnathos presents a pointed ventral projection, as in *Taygetis rectifascia* and *Taygetis fulginia*, but differs from *Taygetis rectifascia* by the absence of the claw-shaped bifid valva apex, and from *Taygetis fulginia* by its stouter valva, with a shorter but wider distal projection of the valva. In additional, *Taygetis servius* is considerably smaller than the other species treated in the present paper.

#### Distribution

(Fig. 8). *Taygetis servius* is known from Baixo Guandú (Espírito Santo), and from Jitaúna (Bahia). It should also be present in Minas Gerais (see below).

#### Taxonomic comments.

*Taygetis servius* stat. n. was described from an unknown number of specimens from Minas Gerais, Brazil, as a form of *Taygetis rectifascia* (Weymer, 1910: 187). Although clearly recognized as such, the illustration of *Taygetis servius* (Weymer, 1910: pl. 46, fig. c [3]) is placed in another plate, separated from the rest of the illustrations of *Taygetis rectifascia* and its forms (Weymer 1910: pl. 45, fig. a [1-2]). The description and the illustration matches exactly a series of five specimens from the states of Espírito Santo and Bahia, Brazil, deposited at the DZUP. Wing shape and pattern, also acknowledged by Weymer, and examination of the genitalia confirms it as a distinct species. The type specimen (or specimens) of *Taygetis servius* is missing, however, type specimens of other species of *Taygetis* described by Weymer in the same fascicle of Die Gross-Schmetterlinge der Erde are housed at the SMT and ZSM collections (i.e. *Taygetis mermeria* f. *crameri* (Weymer, 1910), at SMT, and *Taygetina banghaasi* (Weymer, 1910), at ZSM). However, previous and recent searches for type specimens carried out by G. Lamas, O.H.H. Mielke and the curators of the above cited collections did not produce any specimens (Nekrutenko 2001).

### Taygetis fulginia

d’Almeida, 1922 stat. r.

**Figure 5.**
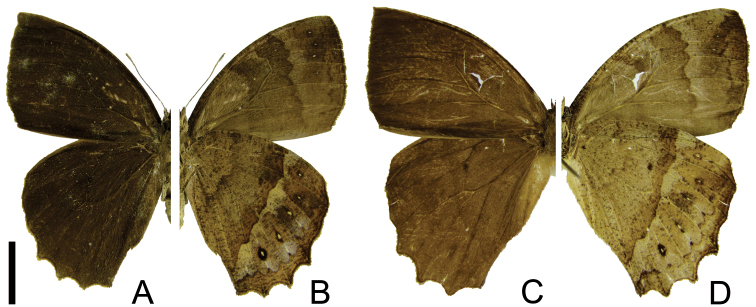
Adults of *Taygetis fulginia* stat. r. **A–B** male **A** dorsal view **B** ventral view **C–D** female **C** dorsal view **D** ventral view. Scale bar = 1 cm.

**Figure 6.**
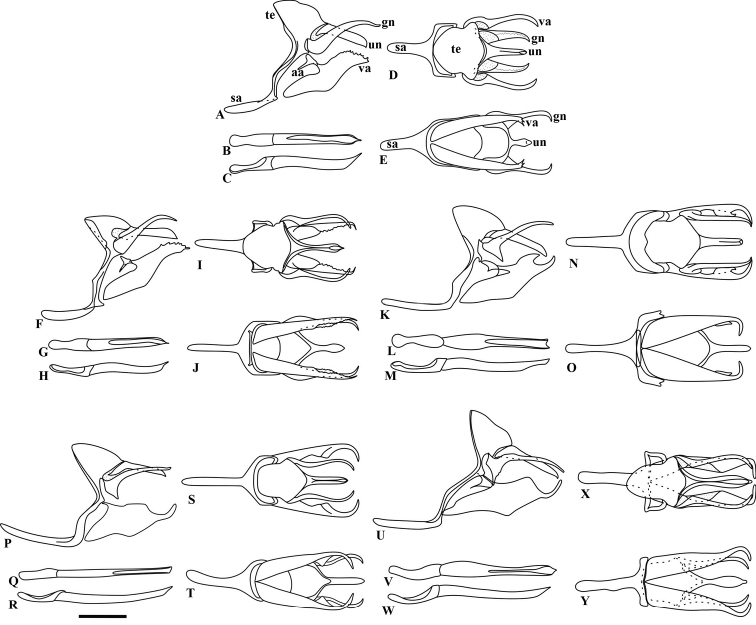
Male genitalia of *Taygetis*. **A–E** *Taygetis drogoni* sp. n. **A** lateral view **B–C** aedeagus: **B** ventral **C** lateral **D** dorsal view **E** ventral view **F–J** *Taygetis ypthima* **F** lateral view **G–H** aedeagus: **G** ventral **H** lateral **I** dorsal view **J** ventral view **K–O** *Taygetis rectifascia* **K** lateral view **L–M** aedeagus: **L** ventral **M** lateral **N** dorsal view **O** ventral view **P–T** *Taygetis servius* stat. n. **P** lateral view **Q–R** aedeagus: **Q** ventral **R** lateral **S** dorsal view **T** ventral view **U–Y** *Taygetis fulginia* **U** lateral view **V–W** aedeagus: **V** ventral **W** lateral **X** dorsal view **Y** ventral view. Abbreviations: **aa** appendix angular; **gn** gnathos; **sa** saccus; **te** tegumen; **un** uncus; **va** valva. Scale bar = 1 mm.

**Figure 7.**
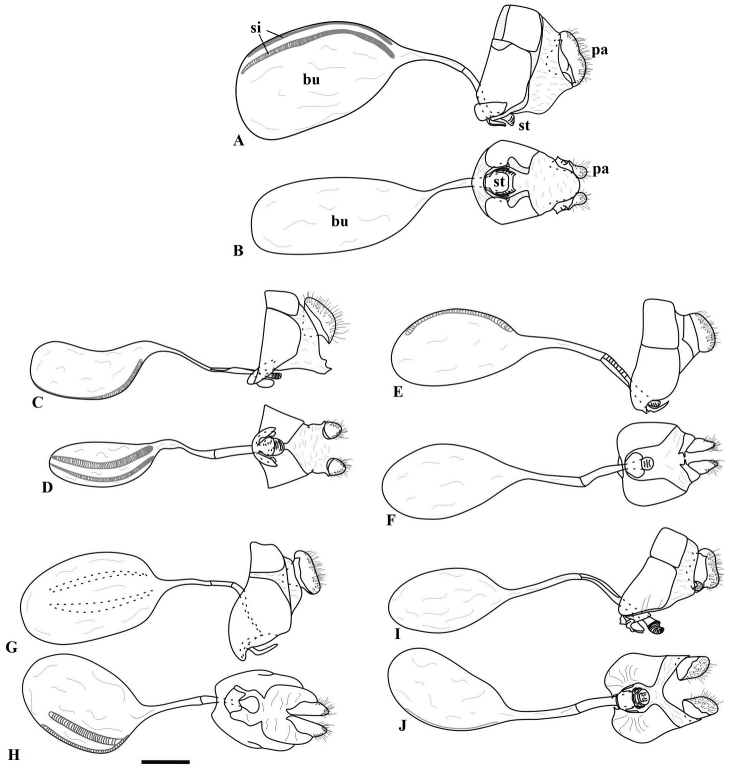
Female genitalia of *Taygetis*. **A–B** *Taygetis drogoni* sp. n. **A** lateral view **B** ventral view **C–D** *Taygetis ypthima* **C** lateral view **D** ventral view **E–F** *Taygetis rectifascia* **E** lateral view **F** ventral view **G–H** *Taygetis servius* stat. n. **G** lateral view **H** ventral view **I–J** *Taygetis fulginia* **I** lateral view **J** ventral view. Abbreviations: **bu** corpus bursae; **pa** papilla analis; **si** signa; **st** sterigma. Scale bar = 1 mm.

Figs 5A–D ; 6U–Y ; 7I, J

#### Type material.

Holotype male with the following labels: /HOLOTYPUS/ *Taygetis fulginia* d’Almeida, 1922 /M(#)/ 30-X-1921 Parada Caramujos, E. F. C. B. [Estação de Ferro Central do Brasil] [Japeri] [22°38'34"S, 43°39'10"W] Estado do Rio [de Janeiro] Ferreira d’Almeida leg. /N°5163/ DZ 27.378/ (DZUP).

#### Additional examined material.

BRAZIL – *Minas Gerais*: **Marliéria** – Parque Estadual do Rio Doce, 250 m, 14-V-1974, 2 males, Ebert leg., ex-coll. Ebert, DZ 26.418, DZ 27.524 (DZUP); 17-V-1974, 1 male, Ebert leg., ex-coll. Ebert,DZ 26.821 (DZUP). *Rio de Janeiro*: **Rio de Janeiro** – Horto Florestal – 01-VIII-1932, 1 female, Gagarin leg., ex-coll. Gagarin,DZ 27.416 (DZUP). *São Paulo*: **Ubatuba** – Parque Estadual da Serra do Mar, Núcleo Picinguaba, 0-100m, 30-IX-2001, 1 male, Brown & Freitas leg. DNA voucher BLU 443 (ZUEC-AVLF).

#### Diagnosis.

*Taygetis fulginia* can be distinguished from *Taygetis ypthima* and other species of the genus by the following characters: in size it is slightly smaller, the forewing is only slightly pointed at the apex, the hind wing has smaller projections at M_3_, CuA_1_ and CuA_2_, similar to *Taygetis servius* stat. n., the dorsal wings lack suffused dark brown bands along the outer margin. The base of the gnathos presents a ventral pointed projection, as in *Taygetis rectifascia* and *Taygetis servius* stat. n., but *Taygetis fulginia* differs from the former by the absence of a developed dorsal projection on the valva, and from the latter by the longer and thinner distal projection of the valva, which is also longer and with a dorsally protruding area in *Taygetis fulginia*.

#### Distribution

**Figure 8.**
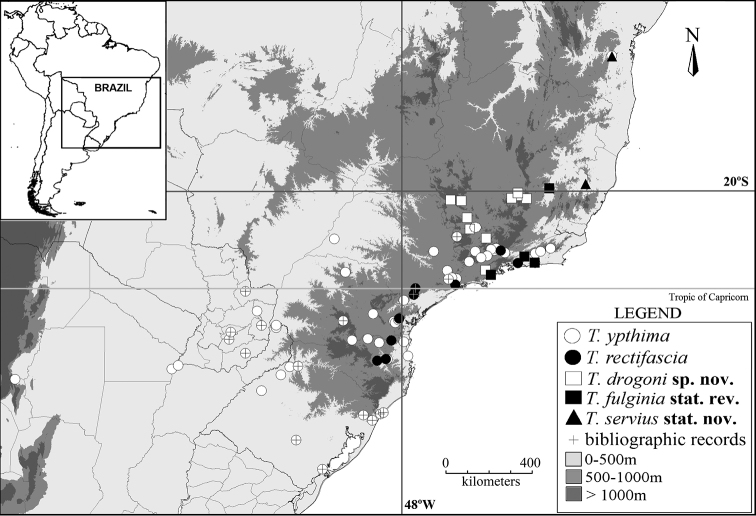
Geographical distribution of the species in the “*Taygetis ypthima* species group”.

(Fig. 8). This species occurs in southeastern Brazil (Minas Gerais, Rio de Janeiro and São Paulo), from sea level to 250m.

#### Taxonomic comments.

The description of *Taygetis fulginia* was based on a single specimen in the D’Almeida collection, now deposited at DZUP (see above). This species was previously considered a synonym of *Taygetis ypthima*, but the morphological study confirms its specific status and indicates closer relationship with *Taygetis rectifascia* and *Taygetis servius* stat. n.

### Key to males and females of the “*Taygetis ypthima* species group”

A combination of wing shape and color pattern permits identification of all five species without dissection, and genitalia of both sexes (not included here but discussed in the text) provide diagnostic characters for all species.

**Table:** 

1	Forewing upper side with suffused dark brown marginal band developed (Figs 1A, C; 2A, C); hind wing with long projections at CuA_1_, CuA_2_ and 2A (Figs 1, 2)	2
–	Forewing upper side with suffused dark brown marginal band reduced (Fig. 3A, C) or absent (Figs 4A, C; 5A, C); hind wing with short projections at CuA_1_, CuA_2_ and 2A (Figs 3, 4, 5)	3
2	Forewing underside submarginal band constricted at M_3_ and reduced or absent in CuA_1_-CuA_2_ (Fig. 1B, D); hind wing underside with the discal line evenly curved and regular; dark post discal line straight and more or less regular (Fig. 1B, D)	*Taygetis drogoni* sp. n.
–	Forewing underside submarginal band not constricted at M_3_ (Fig. 2B, D); hind wing underside with the discal line irregular; post discal line distinctly irregular and not straight (Fig. 2B, D)	*Taygetis ypthima*
3	Forewing apex conspicuously pointed (Fig. 3A–G); forewing upper side with suffused dark brown marginal band reduced (Fig. 3A, C)	*Taygetis rectifascia*
–	Forewing apex rounded (Fig. 4A–D) or slightly pointed (Fig. 5A–D); forewing upper side with suffused dark brown marginal band absent (Figs 4A, C; 5A, C)	4
4.	Ventral hind wing with the proximal border of the submarginal band and post discal line straight and regular, forming an even 2 mm wide creamy white fascia; hind wing with projections at CuA_1_, CuA_2_ and 2A strongly reduced (Fig. 4B, D)	*Taygetis servius* stat. n.
–	Ventral hind wing with the proximal border of the submarginal band and post discal line irregular, forming an irregular creamy white fascia (Fig. 5B, D)	*Taygetis fulginia* stat. r.

## Discussion

The “*Taygetis ypthima* species group” is not part of the genus *Taygetis*, but in fact a clade related to the genus *Pseudodebis* Forster, 1964 (including *Taygetomorpha* L. D. Miller, 2004) in the “*Pseudodebis* subclade” of Matos-Maraví et al. (2013). Four species are certainly part of this clade based on molecular data, namely *Taygetis ypthima*, *Taygetis drogoni* sp. n. (=*Taygetis ypthima* PM10-02 of Matos-Maraví et al. 2013), *Taygetis rectifascia* and *Taygetis fulginia* stat. r., and one additional species was here treated as part of this group based on morphological similarities, *Taygetis servius* stat. n.

The five species treated here can be easily distinguished from one another by wing pattern and genitalia, and two main subgroups can be identified based on morphology - group 1, composed of *Taygetis ypthima* and *Taygetis drogoni* sp. n., and group 2, composed by *Taygetis rectifascia*, *Taygetis fulginia* stat. r. and *Taygetis servius* stat. n. Species in group 2 can be distinguished from those of the group 1 by bearing shorter tails at M_3_, CuA_1_ and CuA_2_, by the lighter dorsal ground color of wings, and by a clear reduction (*Taygetis rectifascia*) or total absence (*Taygetis fulginia* stat. r. and *Taygetis servius* stat. n.) of the suffused dark brown marginal bands. The base of the gnathos in the species of the group 2 presents a conspicuous ventral projection, and there is a longer saccus and aedeagus, and a relatively shorter posterior opening of the aedeagus. Wing color and pattern present conspicuous intraspecific variability in all five species treated here, especially in *Taygetis ypthima* and *Taygetis rectifascia* (Warren et al. 2013), and thorough studies including additional sources of informative characters, e.g. molecular data, might reveal hidden taxonomic diversity.

Although species in the “*Taygetis ypthima* species group” are not part of the genus *Taygetis* (a fact also reinforced by karyological data, see Brown et al. 2007), a taxonomic revision of *Taygetis* and related taxa is not yet available. The lack of such revision is an impediment for eventual species delimitation and the assessment of intraspecific variation as well as the usage of generic synapomorphies. In addition, the diagnosis of many genera within Euptychiina have mostly relied on wing shape and pattern of genitalia (e.g. Forster 1964), making it a hard task to correctly assign species to a genus. As a result, most recent taxonomic rearrangements within Euptychiina are based on DNA sequence data. For example, *Taygetomorpha* L.Miller, 2004 has been recently synonymized with *Pseudodebis* Forster, 1964 (Matos-Maraví et al. 2013), based only on molecular data and larval morphology (Matos-Maraví et al. 2013, Murray 2001a), with no clear adult synapomorphies yet identified.

The present study revealed that morphological characters, such as wing shape and pattern, and male and female genitalia, were efficient to provide clear-cut species delimitation. However, further detailed morphological studies on Euptychiina are highly required to clarify the species and genera delimitations within the subtribe. As clear morphological keys are generated, several monophyletic groups are easily identified using informative synapomorphies (Freitas 2007, Freitas et al. 2010, Zacca et al. 2013). Nonetheless, because of this lack of information, the description of a new genus for the five species treated here would be premature.

## Supplementary Material

XML Treatment for Taygetis drogoni

XML Treatment for Taygetis ypthima

XML Treatment for Taygetis rectifascia

XML Treatment for Taygetis servius

XML Treatment for Taygetis fulginia

## References

[B1] Bellaver JM Iserhard CA Santos JP Silva AK Torres M Siewert RR Moser A Romanowski HP (2012) Borboletas (Lepidoptera: Papilionoidea e Hesperioidea) de Matas Paludosas e Matas de Restinga da Planície Costeira da região Sul do Brasil. Biota Neotropica 12(4): 181-190. doi: 10.1590/S1676-06032012000400019

[B2] Biezanko CM (1960) Satyridae, Morphidae et Brassolidae da zona sueste do Rio Grande do Sul. Arquivos de Entomologia, série A, Escola de Agronomia “Eliseu Maciel”, 12 pp.

[B3] Bonfantti D Di Mare RA Giovenardi R (2009) Butterflies (Lepidoptera: Papilionoidea e Hesperioidea) from two forest fragments in northern Rio Grande do Sul, Brazil. Check List 5(4): 819-829.

[B4] Brown Jr KS Freitas AVL Schoultz BV Saura AO Saura A (2007) Chromosomal evolution of South American frugivorous butterflies in the Satyroid clade (Nymphalidae: Charaxinae, Morphinae and Satyrinae). Biological Journal of the Linnean Society 92: 467-481. doi: 10.1111/j.1095-8312.2007.00872.x

[B5] D’Abrera B (1988) Butterflies of the Neotropical Region, Part 5 Nymphalidae (concl.), Satyridae. Hill House Publishers, Fearny Creek, 190 pp.

[B6] Dolibaina DR Mielke OHH Casagrande MM (2011) Borboletas (Papilionoidea e Hesperioidea) de Guarapuava e arredores, Paraná, Brasil: um inventário com base em 63 anos de registros. Biota Neotropica 11(1): 343-354. doi: 10.1590/s1676-06032011000100031

[B7] Ebert H (1969) On the frequency of butterflies in eastern Brazil, with a list of butterfly fauna of Poços de Caldas, Minas Gerais. Journal of the Lepidopterists' Society 23 (Suppl. 3): 1–48.

[B8] Forster W (1964) Beiträge zur Kenntnis der Insektenfauna Boliviens XIX. Lepidoptera III. Satyridae. Veröffentlichungen der zoologischen Staatssammlung München 8: 51–188, pls. 27–35.

[B9] Freitas AVL (2004) A new species of *Yphthimoides* (Nymphalidae, Satyrinae) from Southeastern Brazil. Journal of the Lepidopterists' Society 58(1): 7-12.

[B10] Freitas AVL (2007) A new species of *Moneuptychia* Forster (Lepidoptera: Satyrinae, Euptychiina) from the highlands of southeastern Brazil. Neotropical Entomology 36(6): 919-925. doi: 10.1590/s1519-566x2007000600014 18246267

[B11] Freitas AVL Peña C (2006) Description of genus *Guaianaza* for “*Euptychia*” *pronophila* (Lepidoptera: Nymphalidae: Satyrinae) with a description of the immature stages. Zootaxa 1163: 49-59.

[B12] Freitas AVL Emery EO Mielke OHH (2010) A new species of *Moneuptychia* Forster (Lepidoptera: Satyrinae: Euptychiina) from central Brazil. Neotropical Entomology 39(1): 83-90. doi: 10.1590/s1519-566x2010000100011 20305902

[B13] Freitas AVL Mielke OHH Moser A Silva-Brandão KL Iserhard CA (2011) A new genus and species of Euptychiina (Lepidoptera: Nymphalidae: Satyrinae) from southern Brazil. Neotropical Entomology 40(2): 231-237. doi: 10.1590/S1519-566X2011000200012 21584405

[B14] Freitas AVL Barbosa EP Santos JP Mielke OHH (in press) Description of the new genus *Atlanteuptychia* for *Euptychia ernestina* (Lepidoptera, Nymphalidae, Satyrinae). Zoologia, Curitiba.

[B15] Giovenardi R Di Mare RA Sponchiado J Roani S Jacomassa FAF Jung AB Porn MA (2008) Diversidade de Lepidoptera (Papilionoidea e Hesperioidea) em dois fragmentos de floresta no município de Frederico Westphalen, Rio Grande do Sul, Brasil. Revista Brasileira de Entomologia 52(4): 599-605. doi: 10.1590/S0085-56262008000400010

[B16] Iserhard CA Romanowski HP (2004) Lista de espécies de borboletas (Lepidoptera, Papilionoidea e Hesperioidea) da região do vale do rio Maquiné, Rio Grande do Sul, Brasil. Revista Brasileira de Zoologia 21(3): 649-662. doi: 10.1590/s0101-81752004000300027

[B17] Iserhard CA Quadros MT Romanowski HP Mendonça MS (2010) Borboletas (Lepidoptera: Papilionoidea e Hesperioidea) ocorrentes em diferentes ambientes na Floresta Ombrófila Mista e nos Campos de Cima da Serra do Rio Grande do Sul, Brasil. Biota Neotropica 10(1): 309-320. doi: 10.1590/s1676-06032010000100026

[B18] Kochalka JA Torres D Garcete B Aguilar C (1996) Lista de invertebrados de Paraguay pertenecientes a las colecciones del Museo Nacional de Historia Natural del Paraguay, In: Romero Martínez O (Ed) Colecciones de Flora y Fauna del Museo Nacional de Historia del Paraguay. Museo Nacional de Historia Natural del Paraguay, San Lorenzo, 69-283.

[B19] Krüger CP Silva EJE (2003) Papilionoidea (Lepidoptera) de Pelotas e seus arredores, Rio Grande do Sul, Brasil. Entomologia y Vectores 10(1): 31-45.

[B20] Lamas G (2004) Nymphalidae. Satyrinae. Euptychiina. In: Lamas G (Ed) Atlas of the Neotropical Lepidoptera. Volume 5A. Checklist: Part 4A. Hesperioidea – Papilionoidea. Association for Tropical Lepidoptera/Scientific Publishers, Gainesville, 217-223.

[B21] Marín MA Peña C Freitas AVL Wahlberg N Uribe SI (2011) From the phylogeny of the Satyrinae butterflies to the systematics of Euptychiina (Lepidoptera: Nymphalidae): history, progress and prospects. Neotropical Entomology 40(1): 1-13. doi: 10.1590/s1519-566x2011000100001 21437476

[B22] Matos-Maraví PF Peña C Willmott KR Freitas AVL Wahlberg N (2013) Systematics and evolutionary history of butterflies in the ‘‘Taygetis clade’'(Nymphalidae: Satyrinae: Euptychiina): Towards a better understanding of Neotropical biogeography. Molecular Phylogenetics and Evolution 66(1): 54-68. doi: 10.1016/j.ympev.2012.09.005 23000820

[B23] Murray DL (2001a) Systematics of neotropical satyrine butterflies (Nymphalidae: Satyrinae: Euptychiina) based on larval morphology and DNA sequence data and the evolution of life history traits. Doctoral dissertation at the Department of Entomology, Graduate Faculty of the Louisiana State University and Agricultural & Mechanical College, Louisiana, 367 pp.

[B24] Murray DL (2001b) Immature stages and biology of *Taygetis* Hübner (Lepidoptera: Nymphalidae). Proceedings of the Entomological Society of Washington 103(4): 932-945.

[B25] Nekrutenko YP (2001) A catalogue of the type specimens of Nymphalidae deposited in the collection of the Staatliches Museum für Tierkunde Dresden (Insecta: Lepidoptera: Rhopalocera). Entomologische Abhandlungen Museum für Tierkunde Dresden 59: 325-403.

[B26] Niculescu EV (1972-1983) L’armure génitale chez les Lépidoptères. Bulletin de la Sociéte Entomologique de Mulhouse (Suppl.), 95 pp.

[B27] Oiticica-Filho J (1946) Sobre a morfologia do penis em Lepidoptera. Boletim do Museu Nacional, Rio de Janeiro, n. s., Zoologia 50: 1-79.

[B28] Paz ALG Romanowski HP Morais ABB (2008) Nymphalidae, Papilionidae e Pieridae (Lepidoptera: Papilionoidea) da Serra do Sudoeste do Rio Grande do Sul, Brasil. Biota Neotropica 8(1): 141-149. doi: 10.1590/s1676-06032008000100017

[B29] Pedrotti VS Barros MP Romanowski HP Iserhard CA (2011) Occurrence of fruit-feeding butterflies (Lepidoptera: Nymphalidae) in a fragment of Araucaria Moist Forest in Rio Grande do Sul State, Brazil. Biota Neotropica 11(1): 385-390. doi: 10.1590/S1676-06032011000100036

[B30] Peña C Lamas G (2005) Revision of the butterfly genus *Forsterinaria* Gray, 1973 (Lepidoptera: Nymphalidae, Satyrinae). Revista Peruana de Biologia 12(1): 5-48.

[B31] Peña C Nylin S Freitas AVL Wahlberg N (2010) Biogeographic history of the butterfly subtribe Euptychiina (Lepidoptera, Nymphalidae, Satyrinae). Zoologica Scripta 39: 243-258. doi: 10.1111/j.1463-6409.2010.00421.x

[B32] Quadros FC Dorneles AL Corseuil E (2004) Ninfalídeos (Lepidoptera, Nymphalidae) ocorrentes no norte da Planície Costeira do Rio Grande do Sul, Brasil. Biociências 12(2): 147-164.

[B33] Ribeiro DB Batista R Prado PI Brown Jr. KS Freitas AVL (2012) The importance of small scales to the fruit-feeding butterfly assemblages in a fragmented landscape. Biodiversity and Conservation 21: 811-827. doi: 10.1007/s10531-011-0222-x

[B34] Santos JP Iserhard CA Teixeira MO Romanowski HP (2011) Fruit-feeding butterflies guide of subtropical Atlantic Forest and Araucaria Moist Forest in State of Rio Grande do Sul, Brazil. Biota Neotropica 11(3): 253-274. doi: 10.1590/s1676-06032011000300022

[B35] Silva ARM Castro CO Mafia PO Mendonça MOC Alves TCC Beirão MV (2012) Borboletas frugívoras (Lepidoptera: Nymphalidae) de uma área urbana (Área de Proteção Especial Manancial Cercadinho) em Belo Horizonte, Minas Gerais, Brasil. Biota Neotropica 12(3): 292-297.doi: 10.1590/S1676-06032012000300028

[B36] Soares A Bizarro JMS Bastos CB Tangerini N Silva NA Silva AS Silva GB (2011) Preliminary analysis of the diurnal Lepidoptera fauna of the Três Picos State Park, Rio de Janeiro, Brazil, with a note on *Parides ascanius* (Cramer, 1775). Tropical Lepidoptera Research 21(2): 66-79.

[B37] Uehara-Prado M Freitas AVL Francini RB Brown Jr KS (2004) Guia das borboletas frugívoras da Reserva Estadual do Morro Grande e região de Caucaia do Alto, Cotia (São Paulo). Biota Neotropica 4(1): 1-5. doi: 10.1590/S1676-06032004000100007

[B38] Warren AD Davis KJ Stangeland EM Pelham JP Grishin NV (2013) Illustrated Lists of American Butterflies. http://www.butterfliesofamerica.com

[B39] Zacca T Mielke OHH Pyrcz TW Casagrande MM Freitas AVL Boyer P (2013) *Stegosatyrus*, a new genus of Euptychiina from the grasslands of neotropical realm (Lepidoptera: Nymphalidae: Satyrinae). Zootaxa 3682(2): 331-350. doi: 10.11646/zootaxa.3682.2.7 25243290

